# Hidden Burden of Hypertension in Türkiye: A Multi-Center DAHUDER World Hypertension Day Study Reveals Gaps in Awareness, Adherence, and Control

**DOI:** 10.3390/jcm15093535

**Published:** 2026-05-06

**Authors:** Hilmi Erdem Sumbul, Ihsan Solmaz, Esref Arac, Ismail Demir, Seyit Uyar, Kamil Konur, Ersin Kuloglu, Ahmed Bilal Genc, Celalettin Kucuk, Kubilay Issever, Nizameddin Koca, Alihan Oral, Ozden Yıldırım Akan, Huseyin Ali Ozturk, Bektas Isik, Fatih Necip Arici, Bercem Berent, Hatice Beyazal Polat, Nazif Yalcin, Deniz Cekic, Selcuk Yaylaci, Ramazan Azim Okyay

**Affiliations:** 1 Department of Internal Medicine, University of Health Sciences, Adana Health Practice and Research Center, Adana 01230, Türkiye; erdemsumbul@gmail.com (H.E.S.); drozturkhuseyinali@gmail.com (H.A.O.); beko44485@gmail.com (B.I.); fatihneciparici803@gmail.com (F.N.A.); 2 Internal Medicine Clinic, Health Sciences University, Diyarbakir Gazi Yasargil Training and Research Hospital, Diyarbakir 21010, Türkiye; ihsan2157@gmail.com; 3 Department of Internal Medicine, Faculty of Medicine, Dicle University, Sur, Diyarbakir 21280, Türkiye; esrefarac@gmail.com; 4 Internal Medicine Clinic, University of Health Science, Izmir Bozyaka Training and Research Hospital, Izmir 35170, Türkiye; drismaildemir22@gmail.com (I.D.); ozdenyldrm@gmail.com (O.Y.A.); 5 Department of Internal Medicine, Antalya Training and Research Hospital, Antalya 07100, Türkiye; seyituyar79@hotmail.com; 6 Department of Internal Medicine, Division of General Internal Medicine, Recep Tayyip Erdogan University Faculty of Medicine, Rize 53200, Türkiye; hatice.beyazalpolat@erdogan.edu.tr; 7 Internal Medicine Clinic, Giresun University Training and Research Hospital, Giresun 28200, Türkiye; ersinkuloglu.28@hotmail.com (E.K.); kubilayissever@gmail.com (K.I.); 8 Department of Internal Medicine, Faculty of Medicine, Sakarya University, Sakarya 54290, Türkiye; ahmedbgenc@gmail.com (A.B.G.); decekic@gmail.com (D.C.); selcukyaylaci@sakarya.edu.tr (S.Y.); 9 Department of Internal Medicine, Istanbul University Istanbul Faculty of Medicine, Istanbul 34093, Türkiye; celalettinkucuk@yahoo.com; 10 Department of Internal Medicine, Bursa Faculty of Medicine, Bursa State Training and Research Hospital, University of Health Sciences, Bursa 16059, Türkiye; nkoca@yahoo.com (N.K.); nazifyalcin16@gmail.com (N.Y.); 11 Department of Internal Medicine, Faculty of Medicine, Biruni University, Istanbul 34295, Türkiye; dr.alihanoral@gmail.com; 12 Department of Internal Medicine, Viransehir State Hospital, Sanliurfa 63700, Türkiye; bercemberent@hotmail.com; 13 Public Health Department, Adana Medical School, University of Health Sciences, Adana 01790, Türkiye; razim01@gmail.com

**Keywords:** hypertension, awareness, blood pressure, cardiovascular risk, Türkiye, cross-sectional study, World Hypertension Day, medication adherence

## Abstract

**Background**: Hypertension remains the single most important modifiable risk factor for cardiovascular disease, stroke, and premature mortality worldwide. Despite major pharmacological advances, awareness, treatment, and control rates remain globally inadequate, particularly in low- and middle-income settings. Türkiye occupies a unique epidemiological position at the intersection of European and Asian cardiovascular risk patterns, with national surveys documenting a stable hypertension prevalence of approximately 30% but persistent deficits in disease control. **Methods**: This cross-sectional study was conducted on World Hypertension Day (17 May 2025) across nine provinces in Türkiye representing five geographic regions (Marmara, Aegean, Mediterranean, Black Sea, and Southeastern Anatolia). A total of 1967 adult volunteers were enrolled. Blood pressure was measured following standardized protocols and classified per ESH 2023 guidelines. A structured questionnaire captured sociodemographic data, lifestyle factors, medication adherence, and hypertension-related awareness. **Results**: The median participant age was 51 years; 55.4% were female. Screening-detected hypertension (BP ≥ 140/90 mmHg) was identified in 26.6% and above-optimal blood pressure in 41.6%. A prior hypertension diagnosis was reported by 35.1%. Among the 1277 participants without a known diagnosis, 51.1% had above-optimal or hypertension-range blood pressure readings, including 11.6% with screening-detected hypertension-range blood pressure. Independent predictors in multivariate analysis included age (OR = 1.048), male sex (OR = 1.528), BMI (OR = 1.096), and alcohol consumption (OR = 1.536); regular exercise was protective (OR = 0.796). Among known hypertensive patients, only 50% monitored blood pressure regularly, 30% skipped doses, and awareness of renal (40.4%) and visual (30.6%) complications was notably low. **Conclusions**: This large multi-center screening study reveals a substantial proportion of previously undetected hypertension-range blood pressure readings and persistent management gaps in a volunteer-based Turkish community sample. The observed screening rate below national prevalence averages likely reflects a healthy volunteer effect inherent to this study design. World Hypertension Day offers an effective framework for simultaneous multi-center screening. Targeted interventions should address non-cardiovascular complication awareness, sodium intake, and medication adherence.

## 1. Introduction

Hypertension is recognized as the single most important modifiable risk factor for cardiovascular disease (CVD), stroke, and all-cause mortality globally. The first World Health Organization (WHO) Global Report on Hypertension, published in 2023, documented that more than one billion adults worldwide are affected, yet only 54% are diagnosed, 42% receive treatment, and a mere 21% achieve adequate blood pressure control [1].

Globally, the burden of hypertension is not static. While the WHO European region experienced a modest decrease in hypertension prevalence between 1990 and 2019, the WHO Western Pacific and South-East Asia regions recorded a 144% increase in the total number of hypertensive adults over the same period, compared with only 41% in the European and Americas regions [1]. These regional divergences underscore the urgency of context-specific epidemiological surveillance and intervention [2].

Türkiye occupies a unique position at the intersection of European and Asian epidemiological patterns. Cardiovascular disease is the leading cause of mortality in Türkiye despite the country’s comparatively young age structure, with 50% of the population under 30 years [3]. National hypertension surveillance has been anchored by two landmark cross-sectional surveys. The PatenT study (2003, *n* = 4910) established a national hypertension prevalence of 31.8%, with strikingly low awareness (40.7%), treatment (31.1%), and control rates (8.1%) [4]. The PatenT 2 study (2012, n = 5437) documented a stable prevalence of approximately 30% but notable improvements in awareness (54.7%), treatment (47.4%), and control (28.7%) [5]. These improvements, while encouraging, still leave the large majority of hypertensive Turks without adequate blood pressure control, underscoring the persistent challenge of translating awareness into effective management.

The epidemiological picture is further complicated by Türkiye’s parallel non-communicable disease burden. The TURDEP-II study demonstrated that diabetes prevalence increased by 90% over 12 years, with hypertension present in 31.4% of its study population [6]. The co-occurrence of hypertension, diabetes, and obesity creates a compounding cardiometabolic risk burden that demands integrated surveillance strategies. Data from the CREDIT study similarly documented a hypertension prevalence of 32.7% in a nationwide chronic kidney disease registry [3], linking uncontrolled hypertension to a spectrum of end-organ complications beyond cardiovascular disease.

Beyond prevalence, the quality of hypertension management among diagnosed individuals is of critical concern. Medication non-adherence is a major driver of treatment failure, with reported global rates of antihypertensive non-adherence ranging from 30% to 40% [7]. Forgetfulness has been consistently identified as the leading cause of unintentional non-adherence across diverse populations [8,9], and persistence with antihypertensive therapy declines substantially over time [7]. Home blood pressure measurement has demonstrated superior predictive value for cardiovascular outcomes compared to office blood pressure [10,11], and has been increasingly endorsed by major international guidelines and the HOPE Asia Network consensus framework [12,13].

Yet hypertension awareness encompasses more than medication adherence and self-monitoring. A comprehensive understanding of the range of complications—including renal disease, visual impairment, and cognitive decline—is integral to motivating sustained preventive behavior. Studies have documented marked asymmetries in patient knowledge, with cardiovascular complications (stroke, heart attack) far better recognized than end-organ damage to the kidneys and eyes [14,15,16].

World Hypertension Day (WHD), observed on May 17 each year, was established by the World Hypertension League to promote global awareness and screening. Beyond its public health role, WHD offers a unique logistical framework for simultaneous, standardized multi-center data collection in a single day.

The present study was therefore designed as an opportunistic, community-based screening initiative utilizing WHD 2025—rather than a population-based prevalence survey—to conduct a simultaneous multi-center assessment across nine provinces spanning five of Türkiye’s major geographic regions. Our objectives were to: (1) determine the proportion of above-optimal and hypertension-range blood pressure readings in this geographically diverse volunteer sample; (2) identify demographic and behavioral risk factors independently associated with above-optimal blood pressure using multivariate logistic regression; and (3) comprehensively assess medication adherence, self-monitoring practices, and complication awareness among known hypertensive patients. This study is the first to demonstrate the feasibility of a synchronized, single-day, multi-regional hypertension surveillance model using WHD as a structured epidemiological platform.

## 2. Materials and Methods

### 2.1. Study Design and Setting

This was a cross-sectional, multi-center observational study conducted on World Hypertension Day, 17 May 2025. Data collection was performed simultaneously across nine provinces in Türkiye: Istanbul, Bursa, and Sakarya (Marmara region); İzmir (Aegean region); Antalya and Adana (Mediterranean region); Giresun and Rize (Black Sea region); and Diyarbakır (Southeastern Anatolia region). This framework encompassed approximately 70% of Türkiye’s geographic regions, representing both coastal and inland populations with varying socioeconomic and environmental exposures.

### 2.2. Participants

Consecutive volunteers aged ≥18 years presenting to participating centers on WHD were eligible for inclusion. Participants with incomplete questionnaire data or missing blood pressure measurements were excluded. A total of 1967 participants were enrolled. As participation was entirely voluntary, a healthy volunteer effect may be present, potentially leading to underestimation of the true hypertension burden in the general population. The study was approved by the relevant institutional ethical committee (decision no: 15/619), and written informed consent was obtained from all participants in accordance with the Declaration of Helsinki.

### 2.3. Data Collection

Blood pressure was measured using validated, calibrated an Omron M3 blood pressure monitor. Following a standardized protocol, participants rested for at least five minutes in a seated position with feet flat on the floor and the back supported. Trained healthcare professionals automatically measured blood pressure three times in succession on both arms, and the average of these measurements was used as the baseline. Blood pressure was classified using a simplified three-category scheme based on 2023 ESH guideline thresholds: optimal (<120/80 mmHg), above-optimal (120–139/80–89 mmHg, encompassing ESH ‘normal’ and ‘high-normal’ categories), and hypertension (≥140/90 mmHg) [17]. Because blood pressure was measured on a single visit, all BP-based classifications in this paper refer to screening-detected categories rather than confirmed diagnoses.

A structured questionnaire collected data on: (1) sociodemographic characteristics (age, sex, educational attainment); (2) anthropometric measurements (height, weight for BMI calculation); (3) lifestyle behaviors (smoking history, alcohol consumption, weekly exercise frequency); (4) known hypertension diagnosis; and among known hypertensive patients: (5) medication adherence, self-monitoring frequency, medical follow-up regularity, target blood pressure knowledge, and awareness of hypertension-related complications. No response entries were treated as missing data. Complete-case analysis was applied throughout, with missing values excluded via IBM SPSS Statistics version 25.0 (SPSS Inc., Chicago, IL, USA) default listwise deletion; no imputation was performed. Denominators for percentage calculations were adjusted accordingly.

### 2.4. Statistical Analysis

Data were analyzed using SPSS version 26.0 (IBM Corp., Armonk, NY, USA). Normality was assessed using Kolmogorov–Smirnov and Shapiro–Wilk tests; all continuous variables departed significantly from normality (all *p* < 0.001) and are reported as median and range. Categorical variables are presented as frequencies and percentages. Chi-square tests examined associations between categorical variables. Multivariate binary logistic regression identified independent predictors of above-optimal blood pressure. The binary outcome (above-optimal BP ≥ 120/80 mmHg vs optimal BP < 120/80 mmHg) was pre-specified on two grounds: (i) the well-established continuous gradient of cardiovascular risk from BP values ≥ 120/80 mmHg [17]; (ii) the screening-oriented aim of this study—to identify individuals whose BP readings warrant further evaluation rather than to adjudicate guideline diagnoses on a single visit. Category-specific frequencies remain reported separately in Table 1 and Section 3.2. Predictor variables were selected a priori based on established epidemiological evidence. Variables were coded as follows: age (continuous, per year); sex (binary; male = 1, female = 0, reference: female); BMI (continuous, per kg/m^2^); smoking status (binary; never smoked = 0 [reference], current or former smoker = 1); alcohol consumption (binary; never = 0 [reference], any use = 1); exercise (binary; no regular exercise = 0 [reference], any regular exercise = 1); and educational attainment (binary; below university = 0 [reference], university and above = 1). All predictors were entered as covariates; binary variables were dummy-coded with the lower category as the reference. Collinearity was assessed using Variance Inflation Factor (VIF); all values were below 1.23 (range: 1.06–1.23), indicating no problematic multicollinearity. The overall model was significant (χ^2^ = 383.379, df = 7, *p* < 0.001; Nagelkerke R^2^ = 0.248); Hosmer-Lemeshow fit was adequate (χ^2^ = 4.690, df = 8, *p* = 0.790). The robustness of variable dichotomisation was confirmed in sensitivity analyses using ordinal scalar and categorical model specifications (Supplementary Table S1). Post hoc power was assessed using G*Power 3.1.9.7 and confirmed adequate power for the primary comparison. All tests were two-tailed; *p* < 0.05 was considered significant.

## 3. Results

### 3.1. General Characteristics

A total of 1967 participants were enrolled. The median age was 51.0 (18–94) years and median BMI was 27.8 (14.9–64.6) kg/m^2^. Women comprised 55.4%. The largest age group was 40–59 years (39.9%). Overweight and obesity were observed in 38.9% and 33.1%, respectively. Half of participants were physically inactive. Screening-detected hypertension-range BP was identified in 26.6% of participants, above-optimal BP in 41.6%, and optimal BP in 31.7%. Prior hypertension diagnosis was present in 35.1% (Table 1).

### 3.2. Blood Pressure Status by Prior Diagnosis

Among 1277 participants without prior diagnosis, 624 (48.9%) had optimal blood pressure, while 505 (39.5%) had above-optimal readings and 148 (11.6%) had screening-detected hypertension-range readings. Among the 690 with known hypertension, none had optimal blood pressure: 376 (54.5%) had screening-detected hypertension-range blood pressure and 314 (45.5%) had above-optimal readings. Overall, 68.3% of all participants had above-optimal or hypertension-range blood pressure, and of these, 48.6% had previously undetected hypertension-range readings. The association between prior diagnosis and current BP category was highly significant (χ^2^ = 650.507, df = 2, *p* < 0.001).

### 3.3. Independent Predictors for Above-Optimal Blood Pressure

**Figure 1:**
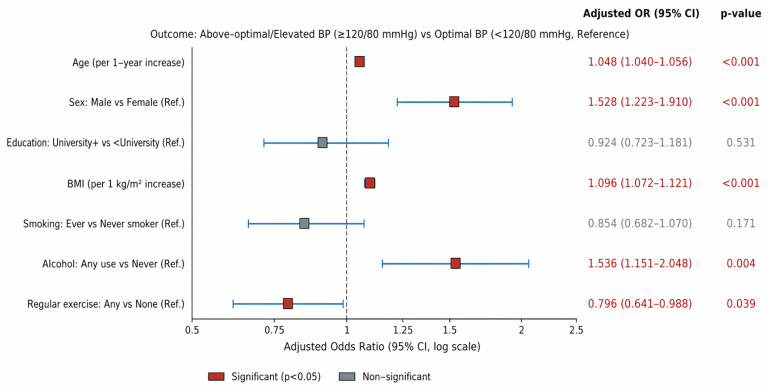
Predictors for Above-Optimal Blood Pressure.

Multivariate logistic regression identified five independent predictors. Age: each additional year was associated with 4.8% higher odds of above-optimal blood pressure risk. (OR = 1.048, 95% CI: 1.040–1.056, *p* < 0.001). Male sex was associated with 52.8% higher odds compared with females (OR = 1.528, 95% CI: 1.223–1.910, *p* < 0.001). BMI was associated with 9.6% increase per 1 kg/m^2^ increment (OR = 1.096, 95% CI: 1.072–1.121, *p* < 0.001). Alcohol consumption was associated with 53.6% higher odds (OR = 1.536, 95% CI: 1.151–2.048, *p* = 0.004), and regular exercise was associated with 20.4% lower odds (OR = 0.796, 95% CI: 0.641–0.988, *p* = 0.039) of above-optimal blood pressure risk. Education and smoking were not independently significant (*p* > 0.05). Results are shown in Figure 1.

### 3.4. Management and Awareness Among Known Hypertensive Patients

**Figure 2:**
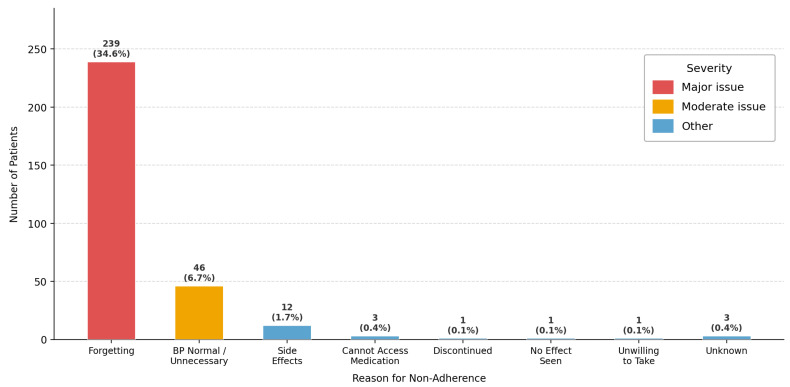
Reasons for Medication Non-Adherence.

**Figure 3:**
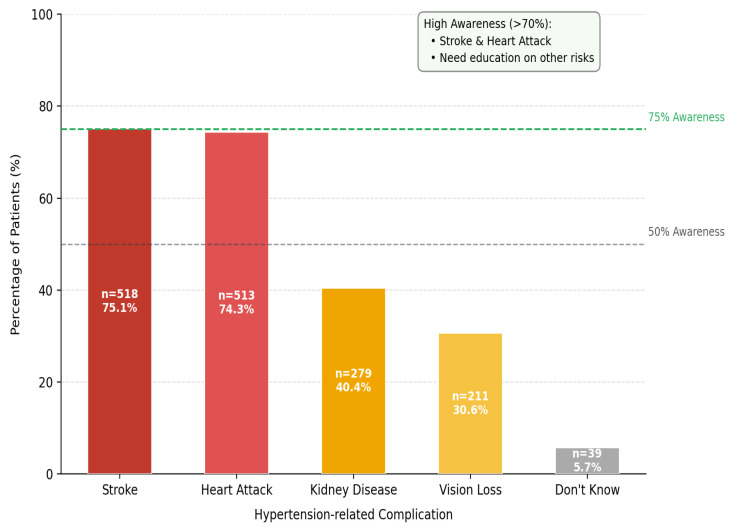
Awareness of Hypertension Complications.

Among 690 known hypertensive patients, 75.9% reported regular medication use; however, 30.0% acknowledged occasionally skipping doses. Only 64.8% attended regular medical follow-up. While 75.4% owned a home blood pressure device, only 50.0% measured regularly. Knowledge of target BP values was present in 79.6%. A striking 70.9% did not perceive their salt intake as excessive (Table 2). The most common reason for medication non-adherence was forgetting (34.6%), followed by perceiving medication as unnecessary when blood pressure normalized (6.7%) and side effects (1.7%). These findings highlight the need for patient education and reminder systems to improve medication compliance (Figure 2). Awareness of complications was high for stroke (75.1%) and heart attack (74.3%), but substantially lower for kidney disease (40.4%) and vision loss (30.6%) (Figure 3).

## 4. Discussion

Before discussing the implications of our findings, it is important to note that the present results represent single-visit, screening-detected blood pressure readings obtained from a self-selected volunteer sample and should not be interpreted as confirmed hypertension diagnoses or population-based prevalence estimates. This multi-center cross-sectional study conducted on World Hypertension Day 2025 across nine Turkish provinces provides a contemporaneous snapshot of hypertension epidemiology, risk factor profiles, and management quality in a geographically diverse community sample. The core findings—the screening-detected hypertension rate of 26.6%, a large burden of previously undetected hypertension-range blood pressure, and substantial deficits in self-monitoring, medication adherence, and complication awareness—carry important implications for cardiovascular disease prevention in Türkiye.

The screening-detected hypertension rate of 26.6% was lower than the approximately 30–32% documented in the population-based PatenT and PatenT 2 surveys [4,5]. However, direct comparison requires considerable caution due to fundamental methodological differences: our study employed voluntary recruitment on a single day versus the population-based random sampling used in PatenT studies; blood pressure classification was based on single-visit screening measurements rather than confirmed diagnoses from repeated visits; and our sample covered five of seven geographic regions whereas PatenT studies achieved nationwide representation. Despite the older age profile of our sample (median 51 years; 32.6% aged ≥ 60 years), which would be expected to inflate hypertension rates, our estimate remained below national figures—most plausibly reflecting a healthy volunteer effect characteristic of voluntary awareness day events [4,5]. Furthermore, the absence of representation from Central and Eastern Anatolia, regions historically associated with higher cardiovascular risk and lower socioeconomic status, may contribute to a modest underestimation relative to the national average [3,5].

The finding that 51.1% of participants without a prior hypertension diagnosis had above-optimal or hypertension-range blood pressure—including 11.6% with hypertension-range readings warranting diagnostic confirmation—is noteworthy and warrants attention. This is consistent with the global diagnosis gap reported by the WHO, which estimated that 46% of hypertensive individuals remain undiagnosed [1]. In the Turkish context, despite improvements documented in PatenT 2, a substantial proportion of the population with above-optimal blood pressure remains undetected [5]. The concurrent above-optimal blood pressure category (39.5% of undiagnosed participants) represents a population at high risk of incident hypertension; prospective data have confirmed that above-optimal blood pressure independently predicts future cardiovascular events and progression to overt hypertension [17].

The identified independent predictors for above-optimal blood pressure are broadly consistent with the existing literature. The age association (OR = 1.048 per year, approximately 60% higher odds per decade) is consistent with well-established progressive arterial stiffening and reduced baroreflex sensitivity [17]. Association with male sex (OR = 1.528) aligns with data from PatenT 2 [5] and TURDEP-II [6], and has been attributed to differences in sex hormone profiles, renal sodium handling, and body fat distribution. Each additional 1 kg/m^2^ of BMI was associated with 9.6% higher odds of above-optimal blood pressure. This finding is consistent with HinT, which reported hypertension incidence rates of 14.2%, 24.5%, and 37.8% for lean, overweight, and obese adults [18], and with other international studies [19]. Although our regression analysis outcome combined above-optimal and hypertension-range readings, the clinically meaningful distinction between these two strata is preserved descriptively in Table 1 and Section 3.2 and should not be obscured by the combined model.

Alcohol consumption was identified as an independent predictor (OR = 1.536). This finding aligns with a large body of evidence documenting a positive, near-linear dose–response relationship between alcohol intake and hypertension risk across both sexes [20,21]. The relatively low overall alcohol consumption rate in our sample (80% never drank) likely reflects cultural norms in Türkiye; however, even the minority who consumed alcohol regularly showed substantially elevated risk.

Regular exercise was significantly associated with lower odds of above-optimal blood pressure (OR = 0.796). This is supported by extensive prospective and interventional evidence. A meta-analysis of longitudinal cohort studies encompassing 330,222 individuals found that achieving minimum physical activity guideline requirements was associated with a 6% reduction in incident hypertension [22]. Leisure-time physical activity has been shown to reduce systolic blood pressure by a clinically meaningful 5.35 mmHg in hypertensive patients [23], and aerobic training is classified as a Class I, Level A recommendation in current hypertension guidelines [17]. The high frequency of physical inactivity in our sample (49.5% never exercised) thus represents a major modifiable population-level risk factor.

The medication adherence data reveal a paradox that resonates with global findings. Despite 75.9% of known hypertensive patients reporting regular medication use, 100% had above-optimal or hypertension-range blood pressure on examination. This dissociation may reflect a combination of self-report bias, suboptimal pharmacological regimens, and genuine adherence failure. However, this should be interpreted cautiously; a public-screening environment, unfamiliar personnel, and the inherent alertness associated with attending a hypertension-awareness event can elicit a white-coat response. In addition, individuals who suspect that their BP is poorly controlled may be more likely to attend WHD events. Globally, antihypertensive non-adherence rates range from 30% to 40% [7], and our observed dose-skipping rate of 30% falls squarely within this range. The dominant reason for non-adherence in our study—forgetfulness—mirrors findings from multiple independent settings [8,9]. Forgetfulness-driven non-adherence is classified as ‘unintentional’ and is particularly amenable to intervention through reminder-based digital tools, simplified once-daily regimens, and single-pill combinations [7,8].

The home blood pressure monitoring data revealed a telling discordance: while 75.4% of known hypertensive patients owned a device, only 50.0% used it regularly. Home blood pressure measurement has been shown to be a stronger predictor of cardiovascular outcomes than office blood pressure [10,11], and its regular use is associated with better treatment adherence and blood pressure control.

The complication awareness findings warrant particular attention. While awareness of cardiovascular complications (stroke: 75.1%; heart attack: 74.3%) was relatively high, awareness of renal disease (40.4%) and vision loss (30.6%) was markedly lower. This pattern is not unique to Türkiye. Abu et al. identified analogous gaps in a US hypertensive population, with one-third unaware that hypertension causes kidney disease [14]. A Tanzanian study documented even lower awareness of renal (32.0%) and eye complications (44.2%) among hypertensive patients [24]. Hypertension is a leading cause of chronic kidney disease, left ventricular hypertrophy, and hypertensive retinopathy [15,16]. The low awareness of these complications may contribute to underestimation of disease severity and reduced motivation for strict blood pressure control and medication adherence. Patient education programs that address the full spectrum of hypertensive end-organ damage—not only cardiovascular events—are urgently needed.

Dietary salt perception represents another critical gap: 70.9% of known hypertensive patients did not consider their salt intake excessive. This perception stands in stark contrast to national dietary data, which reveal that salt intake in Türkiye is nearly two to three times higher than the WHO-recommended maximum of 5 g/day. The WHO 2023 report highlighted that excessive salt intake (≥5 g/day) was responsible for 2 million cardiovascular deaths in 2019, and that potassium-enriched salt substitutes represent a cost-effective blood pressure-lowering strategy [1]. Türkiye has taken legislative steps to reduce dietary salt—mandating reductions in bread and processed food salt content and restricting salt in public cafeterias—and these measures may have contributed to the 11% decrease in hypertension prevalence observed in TURDEP-II between 1998 and 2010 [6]. Nevertheless, patient-level salt perception remains a critical barrier.

### Strengths and Limitations

This study has several important strengths. To our knowledge, it is the first Turkish study to explicitly leverage World Hypertension Day as a simultaneous, multi-center research framework, demonstrating the feasibility and epidemiological value of this approach. The use of a single calendar day for data collection across nine geographically dispersed centers eliminates temporal and seasonal variability in blood pressure measurements. The sample size of 1967 provided adequate statistical power for the primary comparisons. The multi-regional design spanning five geographic zones captures meaningful demographic, climatic, and socioeconomic diversity.

Several limitations must be acknowledged. As a cross-sectional study, causal inferences cannot be drawn. Self-reported lifestyle behaviors are subject to social desirability bias. First and most importantly, the voluntary nature of WHD participation introduces a healthy volunteer effect: Individuals attending WHD screening events tend to be more health-conscious, have better healthcare access, and over-represent older adults already engaged with the health system, while under-representing younger adults, individuals with limited mobility, and residents of regions without participating centers. These factors are expected to bias our estimates of screening-detected above-optimal blood pressure downward; accordingly, findings should be interpreted as descriptive of a volunteer screening sample and not as population-level prevalence estimates. Central Anatolia, Eastern Anatolia, and parts of the Black Sea region were unrepresented, limiting nationwide generalizability. Blood pressure was classified based on a single-visit screening measurement, whereas guideline-concordant hypertension diagnosis requires confirmation on at least two separate occasions [17]; our findings should therefore be interpreted as screening-detected blood pressure categories rather than definitive diagnoses. Finally, the absence of data on antihypertensive regimen complexity, treatment duration, and socioeconomic status limits adjustment for important confounders.

## 5. Conclusions

This large-scale, geographically diverse multi-center study demonstrates that above-optimal blood pressure remains a major screening finding among community-recruited Turkish adults attending WHD events, with a substantial burden of previously unrecognised screening-detected hypertension-range blood pressure requiring diagnostic confirmation, persistent blood pressure elevation among treated patients, and significant deficits in self-monitoring, medication adherence, and awareness of non-cardiovascular complications. The lower screening rate compared with national estimates most plausibly reflects a healthy volunteer effect inherent to the study design rather than true population-level improvement. World Hypertension Day provides an effective framework for simultaneous multi-center screening and should be systematically employed for both epidemiological research and targeted public health intervention. Our findings call for educational programs explicitly addressing renal and visual complication risk, sodium intake reduction, and strategies to mitigate forgetfulness-driven medication non-adherence, alongside the integration of digital self-monitoring tools into routine hypertension care. Opportunistic screening strategies such as WHD may serve as scalable, low-cost public health interventions to identify individuals with undetected above-optimal or hypertension-range blood pressure in community settings.

## Figures and Tables

**Table 1:** Sociodemographic and Clinical Characteristics of Participants (N = 1967).

Characteristics	n (%)
Gender	
Female	1089 (55.4)
Male	878 (44.6)
Age Groups	
<40 years	540 (27.5)
40–59 years	785 (39.9)
≥60 years	642 (32.6)
Educational Status	
Illiterate	184 (9.4)
Primary–High School	1204 (61.2)
University and above	579 (29.4)
Smoking History	
Never smoked	1034 (52.6)
Current smoker	674 (34.3)
Former smoker	259 (13.2)
Alcohol History	
Never	1573 (80.0)
Rarely	260 (13.2)
1–2 times per week	116 (5.9)
Daily	18 (0.9)
Weekly Exercise Status	
Never	974 (49.5)
1–2 days	541 (27.5)
3–4 days	213 (10.8)
5 days and above	239 (12.2)
BMI Categories	
Normal (<25 kg/m^2^)	550 (28.0)
Overweight (25–29.9 kg/m^2^)	765 (38.9)
Obese (≥30 kg/m^2^)	652 (33.1)
Blood Pressure Status *	
Optimal (<120/80 mmHg)	624 (31.7)
Above-optimal (120–139/80–89 mmHg)	819 (41.6)
Hypertension (≥140/90 mmHg)	524 (26.6)
Known History of Hypertension	
No	1277 (64.9)
Yes	690 (35.1)

* Blood pressure classified using a simplified three-category scheme based on 2023 ESH guideline thresholds [17].

**Table 2:** Management and Awareness Among Patients with Known Hypertension (N = 690).

Variable	n (%)
Medication Adherence	
Regular use	524 (75.9)
Non-regular use	58 (8.4)
No response	108 (15.7)
Medication Skipping	
Never skip	370 (53.6)
Sometimes skip	207 (30.0)
No response	113 (16.4)
Regular Medical Follow-up	
Yes	447 (64.8)
No	126 (18.3)
No response	117 (17.0)
Home BP Monitor Ownership	
Available	520 (75.4)
Not available	170 (24.6)
Regular BP Monitoring	
Yes	345 (50.0)
No	345 (50.0)
Last BP Measurement	
Today	168 (24.3)
Within last week	277 (40.1)
Within last month	141 (20.4)
Longer time ago	101 (14.6)
Never measured	3 (0.4)
Knowledge of Target BP	
Knows	549 (79.6)
Does not know	141 (20.4)
Salt Intake Perception	
Thinks it is excessive	143 (20.7)
Thinks it is not excessive	489 (70.9)
Does not know	58 (8.4)

## Data Availability

The original contributions presented in this study are included in the article/Supplementary Materials. Further inquiries can be directed to the corresponding author.

## Supplementary Materials

The following supporting information can be downloaded at: https://www.mdpi.com/article/10.3390/jcm15093535/s1, Table S1. Sensitivity Analysis: Comparison of Three Logistic Regression Model Specifications.

## Abbreviations

The following abbreviations are used in this manuscript:

ESH	European Society of Hypertension
OR	Odds Ratio
CVD	Cardiovascular Disease
WHO	World Health Organization
TURDEP	Türkiye Diabetes, Hypertension, Obesity and Endocrine Diseases Prevalence Study
CREDIT	Chronic Renal Disease In Turkey
HOPE	The Hypertension Cardiovascular Outcome Prevention and Evidence
WHD	World Hypertension Day
BMI	Body Mass Index
CI	Confidence Interval
BP	Blood Pressure
VIF	Variance Inflation Factor
