# Supplementary material for: Hidden Burden of Hypertension in Türkiye: A Multi-Center DAHUDER World Hypertension Day Study Reveals Gaps in Awareness, Adherence, and Control
Source: J Clin Med. 2026 May 6;15(9):3535. doi: 10.3390/jcm15093535 (PMC13163691; doi:10.3390/jcm15093535)
Supplement: Supplementary file 1 [file jcm-15-03535-s001.zip › jcm-4270511-supplementary.pdf]

Table S1. Sensitivity Analysis: Comparison of Three Logistic Regression Model Specifications

Variable	Model 1: Dichotomous		Model 2: Ordinal–Linear		Model 3: Ordinal–Categorical	
	OR (95% CI)	p	OR (95% CI)	p	OR (95% CI)	p
Age (years)	1.048 (1.040–1.056)	**<0.001**	1.048 (1.040–1.056)	**<0.001**	**1.047 (1.040–1.055)**	**<0.001**
Sex (male vs. female)	1.528 (1.223–1.910)	**<0.001**	1.548 (1.237–1.938)	**<0.001**	**1.556 (1.240–1.952)**	**<0.001**
Educational level	0.924 (0.723–1.181)	0.530	0.933 (0.767–1.136)	0.491	—	0.795
High school vs. Primary (ref.)	—	—	—	—	0.916 (0.608–1.381)	0.677
University+ vs. Primary (ref.)	—	—	—	—	0.863 (0.551–1.352)	0.521
Body mass index (BMI)	1.096 (1.072–1.121)	**<0.001**	1.096 (1.071–1.121)	**<0.001**	**1.096 (1.071–1.121)**	**<0.001**
Smoking	0.854 (0.682–1.070)	0.170	0.866 (0.691–1.084)	0.209	0.850 (0.677–1.066)	0.160
Alcohol use	1.536 (1.151–2.048)	**0.004**	1.295 (1.069–1.568)	**0.008**	—	**0.030**
Occasional vs. Never (ref.)	—	—	—	—	**1.458 (1.046–2.031)**	**0.026**
1–2×/week vs. Never (ref.)	—	—	—	—	**1.769 (1.100–2.844)**	**0.019**
Daily vs. Never (ref.)	—	—	—	—	1.111 (0.335–3.681)	0.864[a]
Regular exercise	0.796 (0.641–0.988)	**0.039**	0.886 (0.799–0.982)	**0.021**	—	**0.031**
1–2 days/week vs. None (ref.)	—	—	—	—	0.894 (0.692–1.154)	0.388
3–4 days/week vs. None (ref.)	—	—	—	—	**0.603 (0.428–0.850)**	**0.004**
≥5 days/week vs. None (ref.)	—	—	—	—	0.788 (0.561–1.107)	0.169

OR, odds ratio; CI, confidence interval; ref., reference category.

Model 1 (Dichotomous): alcohol and exercise entered as binary variables (any use vs. never; regular vs. no exercise). Model 2 (Ordinal–Linear): ordinal variables entered as scalar continuous predictors assuming equal spacing between categories. Model 3 (Ordinal–Categorical): ordinal variables entered as indicator contrasts with the lowest category as the reference group; p-values shown for group-header rows in Model 3 are overall Wald chi-square tests (df = 2 for educational level; df = 3 for alcohol and exercise). Significant p-values ($p < 0.05$) are shown in bold.

[a] n = 18 (0.9% of sample). The wide 95% CI (0.335–3.681) reflects insufficient statistical power in this stratum rather than a true absence of effect.
